# Intact perceptual bias in autism contradicts the decreased normalization model

**DOI:** 10.1038/s41598-018-31042-z

**Published:** 2018-08-22

**Authors:** Sander Van de Cruys, Steven Vanmarcke, Jean Steyaert, Johan Wagemans

**Affiliations:** 1 Laboratory of Experimental Psychology, Brain & Cognition, KU Leuven, 3000 Leuven, Belgium; 2 Leuven Autism Research (LAuRes), KU Leuven, 3000 Leuven, Belgium; 3 Department of Child Psychiatry, UPC-KU Leuven, Kortenberg, Belgium

## Abstract

One recent, promising account of Autism Spectrum Disorders (ASD) situates the cause of the disorder in an atypicality in basic neural information processing, more specifically in how activity of one neuron is modulated by neighboring neurons. The canonical neural computation that implements such contextual influence is called divisive (or suppressive) normalization. The account proposes that this normalization is reduced in ASD. We tested one fundamental prediction of this model for low-level perception, namely that individuals with ASD would show reduced cross-orientation suppression (leading to an illusory tilt perception). 11 young adults with an ASD diagnosis and 12 age-, gender-, and IQ-matched control participants performed a psychophysical orientation perception task with compound grating stimuli. Illusory tilt perception did not differ significantly between groups, indicating typical divisive normalization in individuals with ASD. In fact, all individuals with ASD showed a considerable orientation bias. There was also no correlation between illusory tilt perception and autistic traits as measured by the Social Responsiveness Scale. These results provide clear evidence against the decreased divisive normalization model of ASD in low-level perception, where divisive normalization is best characterized. We evaluate the broader existing evidence for this model and propose ways to salvage and refine the model.

## Introduction

In recent years several neurocomputational theories of Autism Spectrum Disorders (ASD) appeared. In contrast to the traditional neurocognitive accounts of ASD, these models enable more specific, model-based predictions of differences in perceptual or cognitive performance between people with and without ASD^1–3^. One such model is derived from a neural population coding model on divisive normalization in the early visual cortex^3^. Divisive normalization (DN) is a so-called canonical neural computation, to explain nonlinear responses in the primary visual cortex, and assumed to be implemented by neural circuits throughout the cortex^4^. It is a ratio (Eq. 1) of the response of a single neuron to the pooled activity of multiple neurons, called the suppressive or normalization pool. It provides a computationally specified way in which the activities of neighboring neurons can be taken into account in determining a given neuron’s outputs. Through normalizing its activity by the average activity in the surrounding neurons, a neuron can adapt its dynamic response range to the current input statistics (gain control), in order to continuously maximize its sensitivity to the variable, natural sensory input. A corollary of this is that responses become robust against (or invariant to) changes in some stimulus dimensions (e.g. different contrast levels). It also entails a form of redundancy reduction, given that responses can be garnered toward information that is not conveyed by neighboring neurons. Apart from gain control, invariance and redundancy reduction, DN can be a mechanism for perceptual and cognitive phenomena that rely on contextual modulation at different levels in the cortex. For example, in low-level perceptual regions, DN has been used to model surround suppression^5^ and binocular rivalry^6^. Finally DN also contributes to winner-takes-all competition among responses to multiple stimuli, in that sensitivity to stimuli eliciting only weak responses is strongly reduced due to divisive normalization, whereas sensitivity to stimuli evoking large responses remains high^7^. This puts DN at the center of the biased competition model of attention.

**Figure 1:**
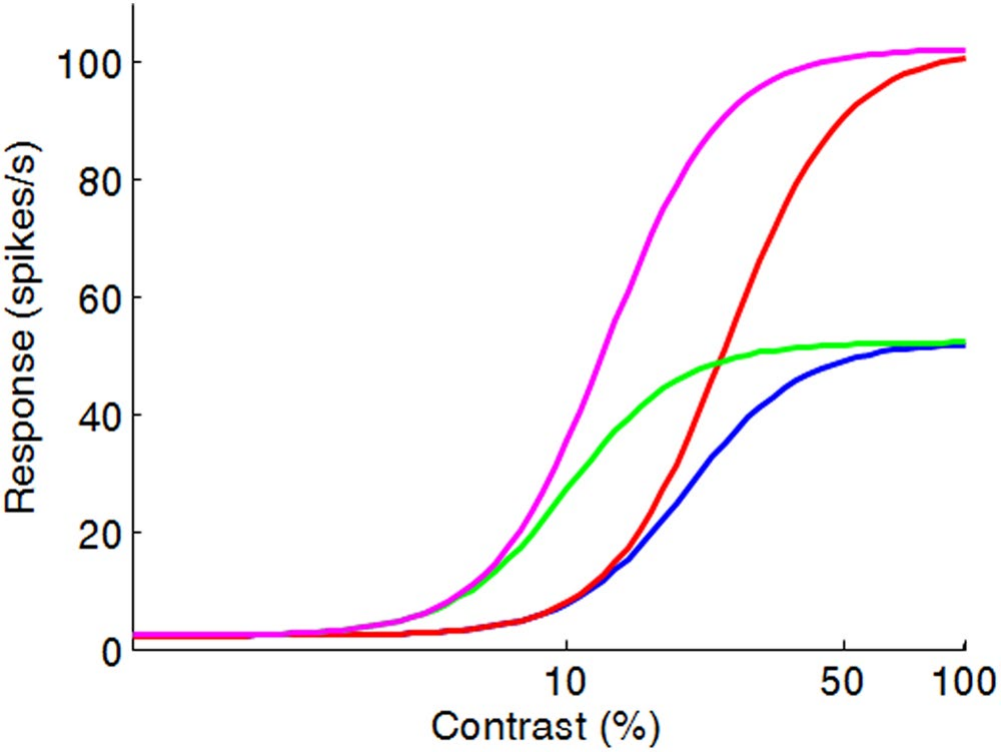
Contrast response function of a V1 cell with *r*_*max*_ = 50 Hz, *c*_50_ = 20% (Michelson contrast), and κ = 1 (blue). When κ is set to 0.5, the maximum response rate is increased to 100 Hz (red). When *c*_50_ decreases to 10%, the contrast response functions shift to the left (green and purple).

This list of proposed roles makes clear why DN is a promising target for explaining neurocognitive alterations in ASD. The key symptoms of ASD are extensive problems in social interaction and communication and a tendency to engage in repetitive and stereotyped behaviors or interests. However, sensory symptoms (e.g. hyper- or hyporeactivity to sensory input) are also considered to be characteristic of the disorder. Certainly, a lack of sensory gain control, of contextual modulation, of redundancy removal, or of attentional control could contribute to the cognitive and perceptual atypicalities found in ASD. Hence, there is great potential for a computational account of ASD that situates the origin of behavioral and perceptual problems in reduced divisive normalization. Rosenberg *et al*.^3^ proposed such a model. They start from the (still controversial) hypothesis that the symptoms of autism are linked to an increased ratio of neural excitation to inhibition, which would result in a reduced impact of the inhibitory or suppressive pool in the DN equation. As is clear from Eq. 1, decreased DN can arise in two different ways. By way of illustration, consider a population of V1 neurons differing in selectivity for orientation (or scale, or position). The contrast response function of a given neuron *i* can be described by the Naka-Rushton equation^8,9^:

1 $$\overline{{r}_{i}}={r}_{0}+{r}_{max}\frac{{[c\ast {f}_{i}(s)]}^{2}}{{c}_{50}^{2}+\kappa {[c\ast {g}_{i}(s)]}^{2}}$$

where *r*_0_ reflects the spontaneous neural discharge (in Hertz) and *r*_*max*_ is the maximum response rate. The numerator of the equation denotes the linear response of a neuron to a stimulus with contrast *c* and orientation *s*, where *f* is a von Mises orientation tuning function (This linear response is amplified by an exponent p to introduce an accelerating non-linearity). The denominator of the equation consists of a semi-saturation constant *c*_50_ which determines the horizontal shift in the response function (see Fig. 1), and the divisive normalization signal, which reflects the linear response of an inhibitory gain control pool of neurons. The orientation tuning function of the gain control pool, *g*, is identical to *f*, except that the orientation bandwidth of *g* is typically much broader. Due to this normalization signal, the response of a neuron *i* to a stimulus with a particular contrast and orientation is inhibited by the responses of neurons in the suppressive pool which respond to a much broader range of orientations. This causes the response of this neuron *i* to saturate at contrasts above *c*_50_. The kappa (*κ*) parameter scales the extent to which the response of a neuron *i* is suppressed by the suppressive pool. It determines to what extent the neighboring neurons influence the neuron’s responses. This parameter equals 1 in the standard contrast normalization model. Reduced DN is thus the result of either a decrease in the semi-saturation constant *c*_50_ or a decrease in the scaling parameter κ (Fig. 1).

In their paper, Rosenberg *et al*. argue for a reduction in the kappa parameter in ASD (specifically a 25% reduction in *κ*) and show that such a model qualitatively matches behavioral data of three recent studies. For example, Foss-Feig, Tadin, Schauder, and Cascio^10^ recently presented drifting gratings of different contrasts and sizes to individuals with ASD and matched controls, who were to detect the motion direction. For gratings of high contrast, performance declined with grating size, indicative of spatial suppression (i.e., divisive normalization) by a pool of neighboring neurons, however detection of individuals with ASD was systematically better across all sizes. For stimuli of small size and contrast, the difference in performance between ASD and controls disappeared, consistent with negligible spatial suppression in this condition (little power in the denominator *c*, hence little impact of differing *κ*’s).

**Figure 2:**
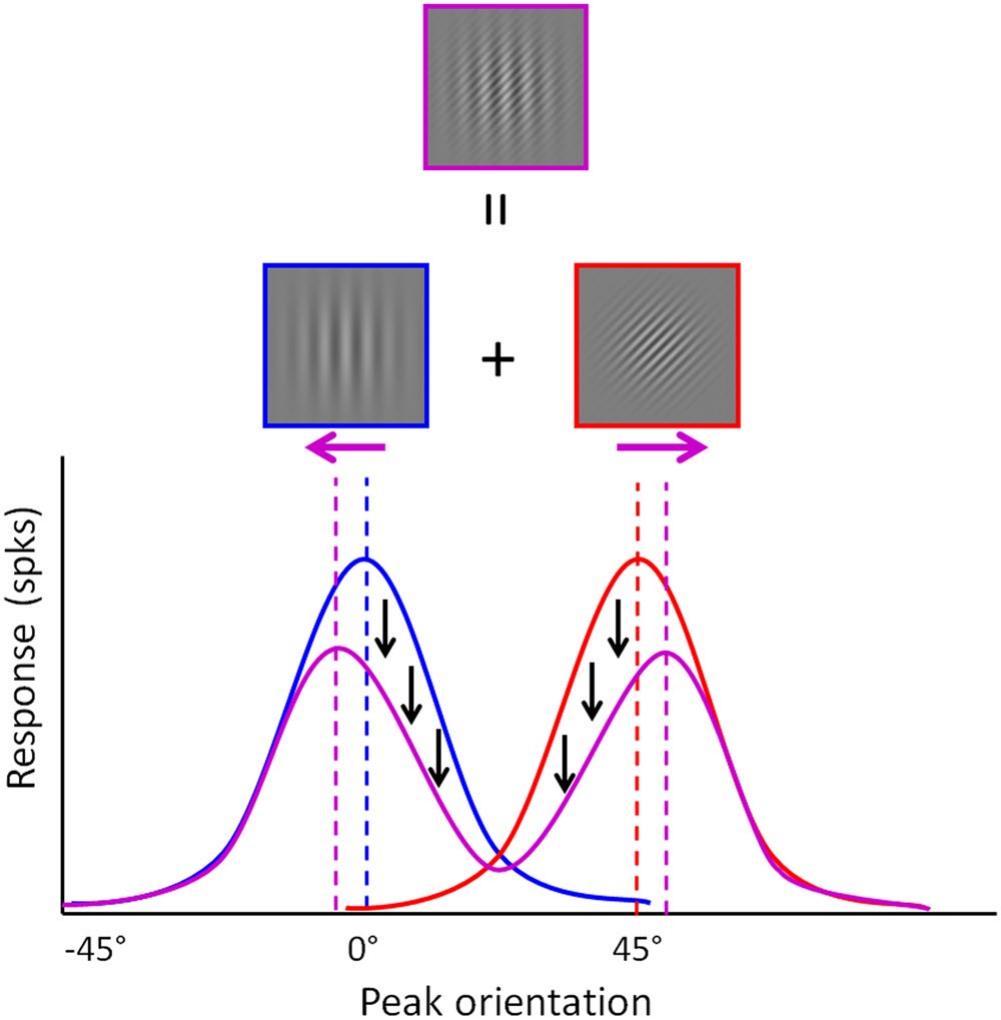
Population response of V1 orientation-sensitive neurons to a sine-wave Gabor grating which is oriented along the vertical axis (blue distribution) and a grating which is oriented clockwise at 45° relative to the vertical axis (red distribution). The purple distribution represents the bimodal population response to a compound stimulus consisting of both gratings.

While Rosenberg *et al*.’s^3^ paper is purely post hoc, the value of their proposal is that it allows us to formulate several straightforward predictions on performance in low-level perception (basic contrast and orientation perception, as well as adaptation). Here, we want to test one of these basic predictions (see also^11^), namely that cross-orientation suppression, as measured by illusory tilt, will be reduced in ASD. In cross-orientation suppression the response of a V1 cell to a target grating at its preferred orientation is inhibited by the superposition of a second grating with a different orientation than the target grating (see Gabor patches in Fig. 2). This physiological finding has been linked to the simultaneous orientation illusion^12,13^: the orientation of the target grating will be perceived as being further away from the orientation of the second grating than it actually is (hence called a ‘repulsive’ effect, see^14^). This would be a direct consequence of divisive normalization: the responses of V1 neurons to the target grating will be suppressed by a broadly-tuned gain control pool of neurons which respond most strongly to the target grating orientation, but which also respond to the neighboring orientations. The addition of a second grating would cause suppression by an additional gain control pool of neurons responding to orientations to which the first gain control pool responds as well (Fig. 2). This will result in more inhibition of neural responses to orientations which are close to the orientation of the added grating.

In the current study, we opted for the superimposed stimulus format (‘overlay suppression’) instead of the surround suppression format because the former has been shown to produce the strongest levels of suppression^15^ and has been most straightforwardly connected to divisive normalization^16^. Indeed, surround suppression is thought to involve more than simple normalization and is found to take place later than overlay suppression^15,17^. As such, overlay suppression seems the purest way to test the DN model of autism.

Several previous studies examined simple (singular) orientation discrimination in ASD, and found either typical^18,19^ or improved^20,21^ orientation discrimination. However, a hitherto untested prediction is that, due to reduced divisive normalization, the “distortion” of orientation perception by context (the added grating) is less pronounced or absent in ASD. This prediction is tested in the present study.

## Results

11 young adults with ASD and 12 age-, gender- and IQ-matched typical individuals had to compare the vertical component of two gratings: one of the stimuli was a single sine-wave grating (blue in Fig. 2) and the other was a compound sine-wave grating consisting of two orientation components (purple in Fig. 2). On each trial, participants judged which interval contained the grating that was most tilted counterclockwise. At any trial, one of the two gratings was varied around the vertical and the other was vertical (see Methods). Our results indicated that there was no group difference in overall accuracy on this orientation perception task (M_ASD_ = 0.69, M_TD_ = 0.73, z = 0.23, p = 0.81). Furthermore, we also did not observe a significant correlation between orientation performance and autism traits as measured by the Dutch version of the Social Responsiveness Scale for adults (SRS-A: r = −0.18, p = 0.4^22,23^) nor between orientation performance and Full-Scale IQ (r = 0.005, p = 0.98). In line with their diagnosis, participants with ASD did score significantly higher than the matched control participants on the SRS-A (t_21_ = 3.41, p = 0.003; see Table 1).

**Table 1:** Overview of the average group-level scores on descriptive measures.

Variables	TD participants	ASD participants	T-test result	Group-level difference?
Age^a^	21.33 (3.77)	24 (3.35)	t_21_ = 1.79, p = 0.09	No
Full scale IQ^a^	109.91 (8.37)	109.09 (6.71)	t_21_ = −0.26, p = 0.80	No
Verbal IQ^a^	111.67 (9.84)	110.45 (7.87)	t_21_ = −0.84, p = 0.40	No
Performance IQ^a^	108.17 (14.17)	106.18 (9.6)	t_21_ = 0.54, p = 0.60	No
SRS (overall)^b^	51.33 (7.70)	65.18 (11.54)	t_21_ = 3.41, p = 0.003	ASD > TD

All tests are two-sample two-tailed t-tests (comparison ASD group and matched TD group). ^a^No group differences on age or IQ, given that groups (ASD and TD) were matched on these variables. ^b^We found an expected significant main effect of Group on overall SRS-A score and subscale scores, with higher scores in the ASD group compared to the TD group.

**Figure 3:**
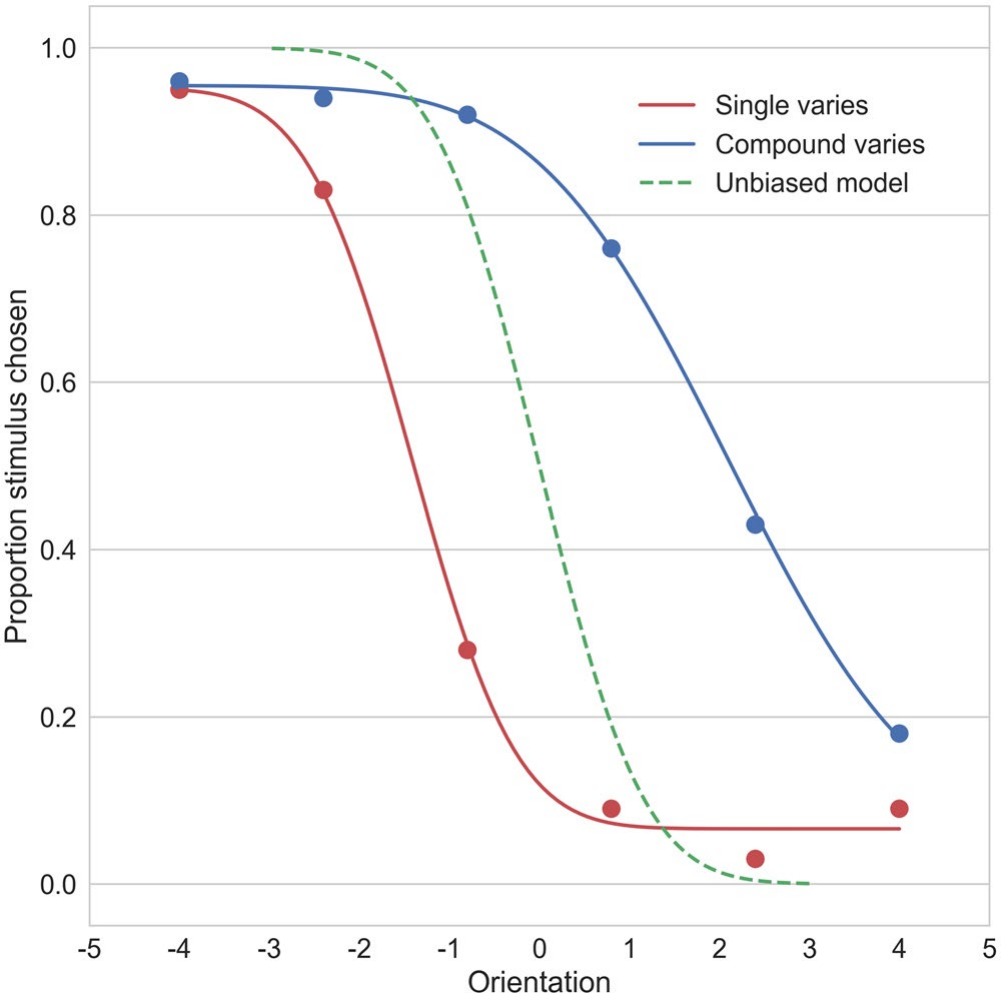
A representative example of psychometric function fits for one participant (with ASD). Dots represent actual data. The Y-axis represents the proportion of trials that the grating was chosen (either compound or single) in those trials that this grating varied. The red line is the fit for the “single varies” condition, the blue for the “compound varies” condition. Green dashed line gives the expected performance of an unbiased participant (no cross-orientation suppression), so with PSE = 0 and slope equal to the slope of the “single varies” condition.

Our main interest lies not in the overall performance, but in the presence and extent of the biases caused by cross-orientation suppression in both groups. Trials were split according to which grating varied (compound or single) and performance was fitted with psychometric functions (two fits per individual). These provided us with a bias parameter which indicates the horizontal shift of the function and, when taken at the 50% point, can be interpreted as the point of subjective equality (PSE): the orientation at which both gratings perceptually look the same. Deriving the PSEs for the ‘single varies’ and ‘compound varies’ conditions is just two different ways of estimating how off-vertical a vertical target grating is perceived when presented in the context of the superimposed 45 degree grating. We also obtained the slope parameter for every fit which signifies the sensitivity to orientation deviations (proportional to the psychometric threshold). Figure 3 shows example fits for the two conditions for a participant with ASD. As expected, a substantial bias would be expressed in both curves as an horizontal shift away from the zero point (unbiased, veridical perception), albeit in opposite directions. Because the “single varies” and “compound varies” conditions are basically two slightly different ways to measure the same thing (namely illusion strength), we averaged over these conditions (i.e. calculated the half-difference between the PSEs of the two curves for each participant). The resulting measure of illusion strength was used as dependent variable in the statistical tests.

**Figure 4:**
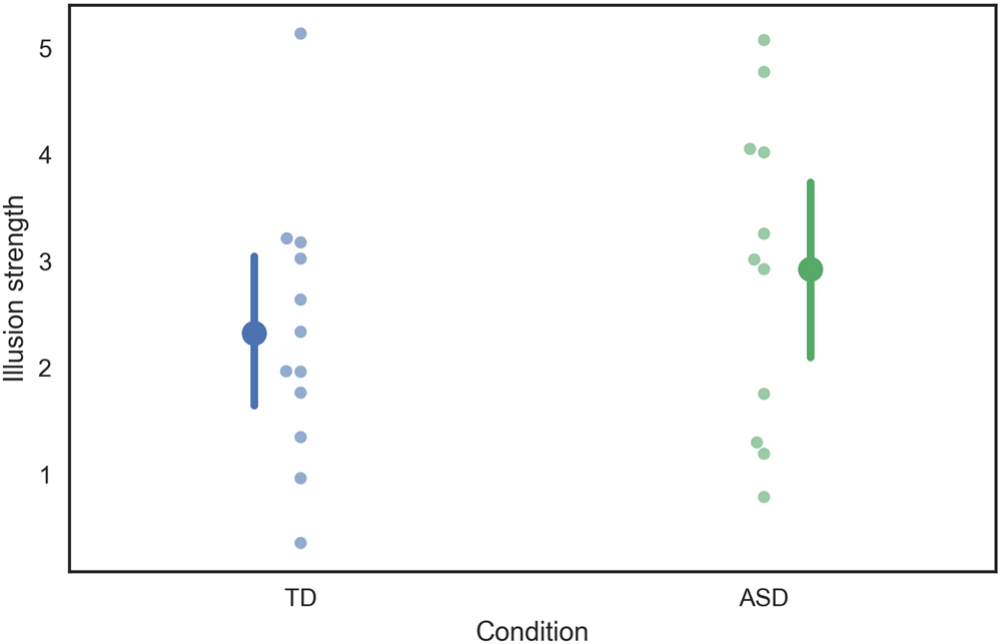
Illusion strengths indicating the extent of perceived illusory tilt caused by a fixed context grating, for the ASD (green) and TD (blue) group, in the two conditions. Darker dots and errorbars are group averages, with bootstrapped 95% confidence intervals. Lighter dots represent the individual data. Note that very similar biases away from zero (veridical perception) can be found in both groups.

As expected based on typical divisive normalization models, all individuals in the control group showed considerable bias. Indeed, none of the individual-level bootstrapped 95% confidence intervals for the PSE contained zero (i.e., nonbiased, “veridical” perception), for both conditions (“single varies” and “compound varies”). Importantly, this was also the case for all participants in the ASD group, indicating that the cross-orientation suppression was not absent in this group. Hence, the average illusion strength was significantly different from zero in both the TD (t_11_ = t = 6.43, p < 0.001) and the ASD (t_10_ = 6.51, p < 0.001) group. In addition, there was no statistical difference in the illusion strengths between groups (t_21_ = 1.05 p = 0.31), contrary to the reduced DN model. If anything, a slightly stronger (nonsignificant) average illusion strength was found in ASD, as can be seen in Fig. 4. In addition, there were no significant correlations between the participants’ score on the SRS-A or Full-Scale IQ and their illusion strength (SRS-A: Pearson r = 0.13, p = 0.56; IQ: Pearson r = −0.11, p = 0.6). In conclusion, while there is considerable variability between individuals with regard to illusion strength (in both participant groups), the bias itself is consistently present and does not vary with ASD diagnosis (or with ASD traits).

While the slope parameter was not of central interest here, we also found no significant group difference here (single varies: t_21_ = 0.35, p = 0.73; compound varies: t_21_ = −1.41, p = 0.17; no correlations with SRS-A or IQ) suggesting that individuals with and without ASD are equally sensitive to orientation deviations.

## Discussion

Contrary to the DN account of ASD, we found no reduced cross-orientation suppression in participants with ASD compared to matched control participants. All participants with ASD showed a level of illusory tilt perception that was comparable to or even stronger than that of typical participants. To the best of our knowledge this is the first direct test of one of the most basic psychophysical predictions of the DN model for autism. To properly evaluate what this means for the DN hypothesis, we first discuss related findings, as well as the limitations of the DN model and of our own study.

Our findings clearly demonstrate that a very basic form of contextual modulation is intact in autism. This contradicts findings on reduced susceptibility to visual phenomena that are caused by context, such as visual illusions^24^, (but see^25^). However, the evidence on contextual modulation in low-level vision, more akin to our study, is incomplete. For example, both increased and decreased collinear facilitation (better detection of a faint grating when flanked by co-aligned gratings) has been reported in ASD^26,27^. To the extent that these modulations are products of divisive normalization, the evidence is far from unequivocal for the overarching DN model by Rosenberg *et al*.^3^. Indeed our findings, as well as a recent study on adaptation to direction of gaze^28^, seriously question such a generalized DN-based mechanism. However, those results do not exclude that contextual modulation may be affected in some instances, whether mediated by a form of divisive normalization or not. For example, the Foss-Feig *et al*.^10^ study, one of the prime pieces of evidence in Rosenberg *et al*.^3^, is interpreted as an effect of attenuated surround suppression, in line with what Flevaris & Murray^29^ reported for individuals with high (but non-clinical) autistic traits (but see^30^ for an example of typical surround suppression). Interestingly, while overlay suppression as investigated in the current study relies on simple contrast normalization^16^, surround suppression is suggested to involve a special normalization mechanism based on redundancy in natural images^15^; (see below). The finding that even different psychophysical measures of surround suppression (duration thresholds in a motion discrimination task as in Foss-Feig *et al*. vs. contrast thresholds in a contrast detection task) are not significantly correlated^31^, further casts doubt on the idea that there is one unified mechanism underlying these phenomena (e.g. a GABA-ergic normalization process that could be impaired in autism). We should also note that none of the three psychophysical findings that Rosenberg *et al*.^3^ were able to fit with their model, have been replicated in ASD so far. In fact, for their third case, which considers the influence of a prior (implemented with DN) that increases the sensitivity for cardinal orientations, there is even a recent non-replication^19^.

Paralleling the behavioral evidence, the neural evidence for reduced DN in ASD is also indirect and inconsistent, up till now. Firstly, the excitatory/inhibitory imbalance theory of ASD, a key inspiration for Rosenberg *et al*.’s model, is considered to be overly simplistic and insufficiently supported by evidence (e.g., see^32,33^). A differentiation needs to be made between different microcircuits in different brain regions, that may require different ratios of inhibition and excitation. While the homeostatic regulation of circuit activity seems disturbed in ASD, our knowledge of the molecular mechanisms affected and their implications for coding and transmission of information is still very limited. For example, it is still unclear whether autism is linked to enhanced excitatory (glutamatergic) excitation or to reduced (GABAergic) inhibition, while the Rosenberg *et al*.’s DN hypothesis requires the latter to be the case. However, conversely it is far from clear that divisive normalization always relies on GABAergic inhibitory connections^4^. This is especially troubling given that Rosenberg *et al*. propose that reduced divisive normalization can also explain higher level processing differences in ASD, such as increased local perceptual focus or altered social functioning and decision-making. Indeed, individuals with ASD make more consistent, less context-dependent decisions^34^, where this context-dependence can also be modeled with DN^35^. Although interesting, the cortical circuits implementing divisive normalization beyond the primary visual cortex are likely to differ widely^4^: divisive normalization is a canonical computation, but not a canonical cortical microcircuit. This implies that there might not be a straightforward mapping from underlying neurophysiological alterations to aberrant computation in ASD.

While our study speaks against a generalized decreased DN in ASD, a few limitations should be taken into account. Firstly, we only tested DN in low-level vision and cannot exclude the possibility that other products of DN, higher up in the perceptual processing chain, could be affected. Secondly, for pragmatic reasons (these perceptual tasks can be quite taxing) we only tested young adults. There is no reason to expect learning or compensation to have a role in such low-level perceptual capacities, but future studies should consider testing younger participants, possibly by using game-like features to increase engagement. Finally, we only targeted one way in which DN can be reduced (the kappa parameter). If however, the c_50_ component in the denominator (see Eq. 1) is affected instead of kappa, different predictions can be made, which we cannot address with the current task. Specifically, one should expect reduced contrast adaptation in ASD, a reduction of the perceived contrast of a test stimulus caused by prolonged exposure to a high-contrast stimulus^36,37^. Physiological evidence suggests that this is mainly due to a decrease in contrast sensitivity (i.e., an increase in c_50_). Though not the preferred parameter for Rosenberg *et al*., they do leave open the possibility that the semi-saturation constant c_50_ might (also) be affected in ASD. While several studies found reduced adaptation in higher-level perception (e.g. faces or biological motion^38,39^), there are to our knowledge no studies characterizing low-level visual adaptation in ASD.

To conclude, while we report clear evidence against the decreased DN model of ASD, we would not dismiss it completely. As indicated in the introduction, the assumed functions of DN find a good match in the cognitive peculiarities of ASD. Specifically, deficient gain control is, explicitly or implicitly, central to several recent accounts of ASD^2,40,41^. We still see great potential in an explanation that centers on the flexible, context-dependent regulation of gain, rather than the simple, static contextual modulation that is called upon in the current task. An example of this flexible gating of contextual influences can be found in the work of Coen-Cagli, Kohn, and Schwartz^42^. These authors show that it makes little sense for the context (neurons) to indiscriminately modulate a given neuron’s activity, when the extent to which context and (classical) receptive field are part of the same inferred cause (homogenous surface) varies in natural images. Hence, they argue that the strength of the suppression should be tuned flexibly based on a (Bayesian) inference on whether target and surround are actually homogeneous. This generalized, flexible DN model is shown to better fit the activity of V1 neurons than a conventional DN model, when applied to natural images^42^. The inference on whether target and surround activity are caused by a common hidden cause in the input, makes the connection explicit with Bayesian accounts of perception, as well as with perceptual grouping or segmentation. Such a model provides several interesting links with existing work in ASD. Firstly, given that the “weight” of the suppression relies on an extra operation (an inference) it can be assumed to take a non-negligible amount of time for the proper contextual modulation to be effected. Indeed, the formation of global percepts (requiring segmentation and grouping) has been shown not to be impossible, as previous accounts of ASD hypothesized, but rather to require more time to develop^43^. Secondly, inference on whether inputs derive from a common, homogeneous cause in space (a perceptual object) or in time (stationarity vs volatility) are important on all levels of the hierarchy, not just in V1. This acknowledgement links the type of flexible gain control discussed here to the flexible gain regulation thought to be deficient in ASD according to recent predictive processing accounts of the disorder^2,44^. In those models, the weight (or “precision”) of prediction errors needs to be lowered when it is inferred that those prediction errors derive from mere noise (so still the same hidden cause) or while the gain should be increased when they derive from an actual change in the environment (a different hidden cause). While this may emerge as a significant commonality in accounts of ASD, the models in which they are grounded differ considerably in their computational and implementation details. Specifically, the DN model is based on population coding theories and divisive suppression, while predictive processing represents hidden causes by sufficient statistics and assumes subtractive, predictive suppression. More fundamental research is needed to see if these models are compatible, or how we can arbitrate between them. In any case, these computational accounts of ASD will surely spawn interesting empirical research to come.

## Methods and Materials

### Participants

11 adults with ASD and 18 age-, gender- and IQ-matched typical young adults participated in the experiment for a monetary compensation. All were naive to the purpose of the study, had normal intelligence, and normal or corrected-to-normal vision.

All participants from the ASD group were previously diagnosed with a pervasive developmental disorder (Autistic Disorder, Asperger syndrome or PPD-NOS), according to DSM-IV-TR criteria^45^, by a multidisciplinary team. Recruitment was exclusively set up via the Autism Expertise Centre of the University Hospital in Leuven. TD participants were recruited with an online recruitment system and selected based on the matching criteria. The study was approved by the Medical Ethics Commission of UPC-KU Leuven. Before the start of the experiment, written informed consent was obtained from all participants.

The data of 6 participants (all TD) were excluded because of (near) chance performance (<0.55 overall accuracy), leaving the data of 11 young adults with ASD (7 males) and 12 TD adolescents (7 males) to use in further analyses. IQ was estimated using a validated four-subtest (Vocabulary, Similarities, Picture Completion and Block Design) version of the WAIS-III^46,47^. Full descriptive information can be found in Table 1.

### Apparatus

Experiments are conducted in a darkened and sound-proof lab room, with a viewing distance of 60 cm to the monitor (using a chinrest). The stimuli are displayed on a calibrated CRT monitor and generated by the ViSaGe stimulus generator (Cambridge Research Systems, Cambridge, England) which is controlled by MATLAB (MathWorks, Natick, US). The spatial resolution of the monitor was 1024 × 768 pixels with a refresh rate of 118 Hz. The mean background luminance of the screen was equal to 72.5 cd/m^2^. Participants were required to give a binary response by pressing either a top or bottom button on a Cedrus response box (RB-530, Cambridge Research Systems).

### Stimuli

The stimuli were Gabor patches (0.33 degrees of visual angle), created by multiplying a cosine grating with a 2D Gaussian envelope (SD = 0.75°). On each trial, one of the stimuli was a single sine-wave grating (blue in Fig. 2) and the other was a compound sine-wave grating consisting of two orientation components (purple in Fig. 2). The single grating had only one component, which varied in orientation (i.e. small deviations from the vertical axis) and had a spatial frequency of 1.5 cycles per degree. For the compound grating, one component (the target) had a variable orientation around the vertical (the spatial frequency of 1.5 cycles per degree) and the other component was always tilted at 45° clockwise relative to the vertical axis. This means the target component is expected to be perceived as more counterclockwise than it really is. The fixed component had a higher spatial frequency (3.75 cycles per degree) to clearly distinguish it from the task-relevant vertical component. Stimuli were displayed on a gray background (Michelson contrast of 50%). All stimuli had a Michelson contrast of 100%. To create a fully balanced experiment we either varied the single or the compound stimulus around the vertical axis (possible orientations: [−4°, −2.4°, −0.8°, 0.8°, 2.4°, 4°]) in each trial. Each stimulus was presented foveally.

### Procedure

Observers performed a 2-interval forced choice (2-IFC) task. In our piloting, we found the strongest suppression effects with foveal presentations (so using a 2-IFC instead of a 2-alternative forced choice task), although the more systematic tests by Petrov, Carandini, & McKee^15^ showed that eccentricity does not influence overlay suppression much (contrary to surround suppression). Each trial consisted of the presentation of a fixation cross (500 ms), followed by two gratings presented sequentially, each for 200 ms, with an inter-stimulus interval of 800 ms. There was no limit on the allowed reaction time, and no feedback was provided. To make sure the participants understood the task, they were asked to do 10–20 practice trials in the presence of the experimenter, with larger orientation deviations and a slightly longer exposure duration per stimulus (500 ms) than in the actual experiment. The actual experiment consisted of 1,200 trials (6 orientations * 2 compound or single varies * 2 compound in first or second interval * 50 per data point) divided in blocks of 50 trials. Participants received feedback between the different blocks. This feedback indicated how well they performed in the previous block (in percentage), and on how far they were (in percentage) in the entire experiment. The experiment took approximately 90 minutes in total and participants were allowed to take small breaks between the test blocks. In a second part of the testing session, we administered the IQ-test and SRS-A questionnaire.

### Statistical analyses

Two full psychometric functions (cumulative Gaussians) were fitted per participant (see *SI* Fig. 1): a first one plotting the proportion of trials that the compound grating was chosen in trials in which the compound grating varied, and a second one plotting the proportion of trials that the single grating was chosen in the trials in which the single grating varied. We used the maximum-likelihood fitting procedure^48^ in *pypsignifit*^49^. We assessed goodness-of-fit (deviance), and the confidence intervals for the parameter estimates using a parametric Monte-Carlo bootstrap procedure involving 10,000 samples^50^.

In addition to the reported analyses, we confirmed all results with a generalized estimating equation analysis (GEE^51^) on a dataset with all (10,000) simulated parameters per participant to take the uncertainty with regard to the parameter estimates into account.

The code for running the experiment, all the data and the analysis scripts are available online at http://osf.io/8hcfp.

### Ethics approval and informed consent

Medical Ethics Commission of UPC-KU Leuven approved this study. Methods were carried out in accordance with the guidelines and regulations of the ethics commission. Before the start of the experiment, written informed consent to participate was obtained from all participants. Given that both our ASD patients and the typical participants were *adults of normal intelligence*, we did not need additional consent of parents or guardians. Written informed consent to use their data in scientific publications was obtained from all participants, and only de-identified data was used for analysis.

## Electronic supplementary material

Supplementary figure 1

## Data Availability

All data and code is made available on the Open Science Framework with persistent link: https://osf.io/8hcfp/ (as indicated in the main text).
