# Supplementary material for: Intact perceptual bias in autism contradicts the decreased normalization model
Source: Sci Rep. 2018 Aug 22;8:12559. doi: 10.1038/s41598-018-31042-z (PMC6105689; doi:10.1038/s41598-018-31042-z)
Supplement: Supplementary file 1 — Supplementary figure 1 [file 41598_2018_31042_MOESM1_ESM.pdf]

Supplementary information: Intact perceptual bias in autism contradicts the decreased normalization model

Sander Van de Cruys, Steven Vanmarcke, Jean Steyaert, Johan Wagemans

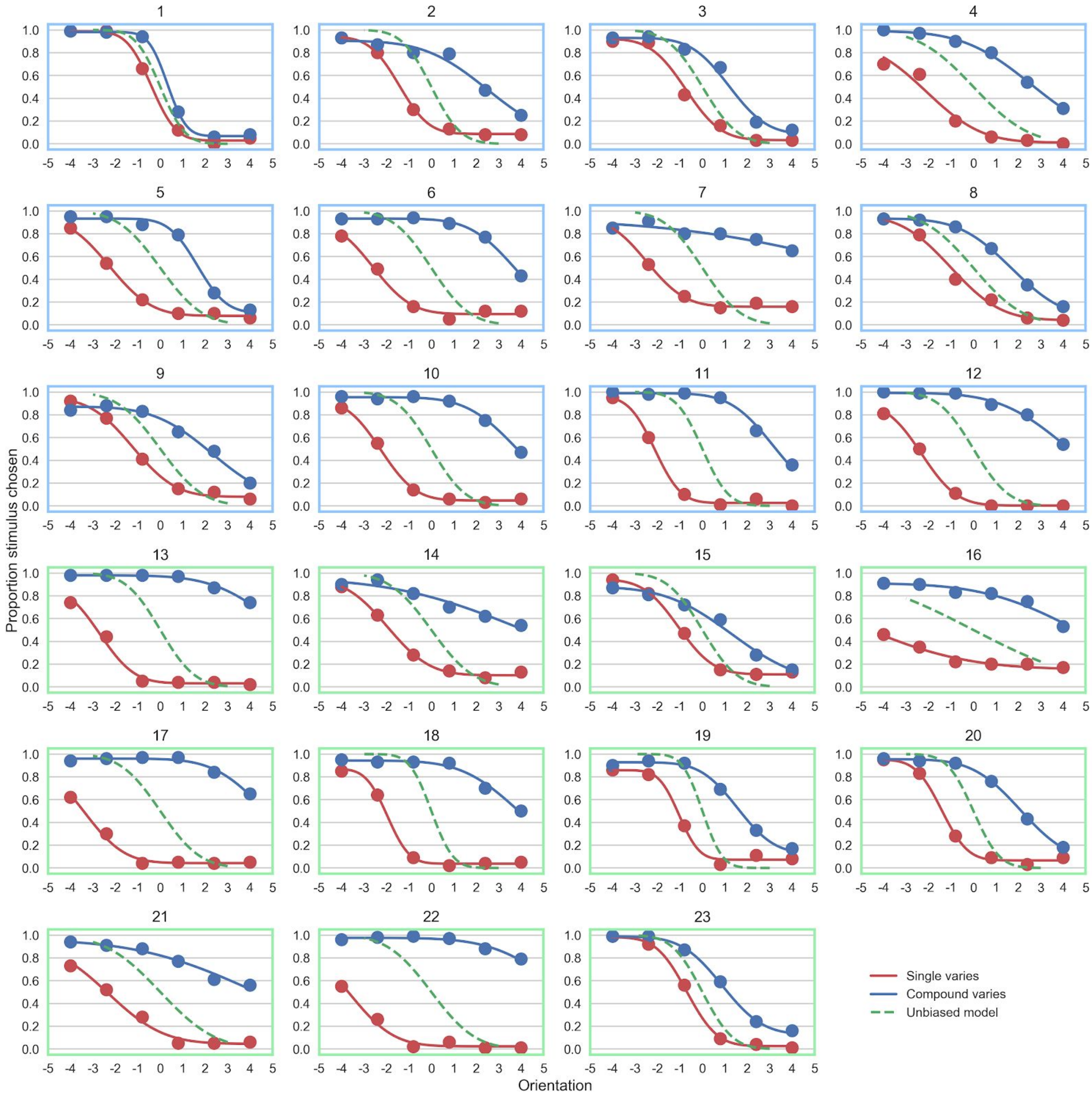

Figure 1: All individual data with their psychometric function fits, for the two conditions (1 plot per individual). Individuals without ASD have light blue frames, those with ASD have light green frames. The unbiased model represents a psychometric function with a PSE of zero and a slope equal to the current participant's slope for the single varies condition.
